# Puerarin alleviates renal ischemia/reperfusion injury by inhibiting apoptosis and endoplasmic reticulum stress via Nrf2/HO-1 pathway

**DOI:** 10.22038/ijbms.2024.80438.17412

**Published:** 2025

**Authors:** Jingsong Wang, Qingyuan Zheng, Zhiyuan Chen, Xiuheng Liu, Shanshan Wan, Lei Wang

**Affiliations:** 1 Department of Urology, Renmin Hospital of Wuhan University, Wuhan, Hubei, 430060, China; 2 Institute of Urologic Disease, Renmin Hospital of Wuhan University, Wuhan, Hubei, 430060, China; 3 Department of Ophthalmology, Renmin Hospital of Wuhan University, Wuhan, Hubei, 430060, China; # These authors contributed equally to this work

**Keywords:** Apoptosis, Endoplasmic reticulum- stress, Nrf2/HO-1 pathway, Puerarin, Renal ischemia/reperfusion- injury

## Abstract

**Objective(s):**

To explore the effects of puerarin on renal ischemia/reperfusion injury and the possible mechanism.

**Materials and Methods:**

The experimental mice were injected with puerarin (50 or 100 mg/kg) per day or equal sterile saline by intraperitoneal injection for one week, and a renal I/R injury model was constructed. HK-2 cells were incubated with puerarin (1 uM and 10 uM) before the H/R model. Immunohistochemistry, immunocytochemistry, and Western blot analysis were used to detect the protein associated with apoptosis and endoplasmic reticulum stress.

**Results:**

Puerarin could improve renal function and attenuate tissue structural damage after renal I/R. Meanwhile, puerarin alleviated apoptosis and endoplasmic reticulum stress by decreasing expression levels of specific biomarkers such as caspase-3, GRP78, CHOP, and p-elF2α/ elF2α in animals and HK-2 cells. The up-regulated expression of Nrf2 and HO-1 protein after puerarin treatment indicated that the Nrf2/HO-1 signaling pathway might mediate the protective mechanism of puerarin against renal I/R.

**Conclusion:**

Our results suggest that puerarin alleviated renal ischemia/reperfusion injury by inhibiting apoptosis and endoplasmic reticulum stress via the Nrf2/HO-1 pathway and offered new insights for preventing and treating renal I/R.

## Introduction

Acute Kidney Injury (AKI) poses a complex clinical challenge with high incidence and mortality rates. It frequently progresses to chronic kidney disease, serving as a significant risk factor for end-stage kidney disease. Renal ischemia/reperfusion (I/R) injury is identified as a primary cause of AKI (1). Despite this, the specific mechanisms of I/R injury remain unclear. Research suggests that apoptosis, oxidative stress, endoplasmic reticulum stress, mitochondrial dysfunction, and ion accumulation are crucial molecular mechanisms (2-4). Whereas AKI and related complications caused by renal ischemia-reperfusion pose a serious threat to human health, it is very valuable to lucubrate its pathophysiological mechanism to provide more effective treatment strategies for renal I/R injury.

Apoptosis emerges as the predominant pathway for cell death in renal tubular epithelial cells during renal I/R. The increased exposure to hypoxia and free radicals disrupts homeostasis in the endoplasmic reticulum, leading to abnormal accumulation of misfolded or unfolded proteins and subsequent endoplasmic reticulum stress, a pivotal process in renal I/R injury (5, 6). Prolonged stress conditions activate the associated apoptosis pathways, culminating in the apoptosis of renal tubular epithelial cells (7).

Puerarin, a naturally occurring isoflavone, has been extensively studied for its pharmacological activities and widespread application in various diseases (8). Numerous studies have confirmed its protective role against I/R injury (9), primarily attributed to its ability to mitigate inflammatory response, oxidative stress, endoplasmic reticulum stress, and apoptosis (10-13). For instance, it was reported that puerarin could alleviate retinal ganglion cell damage induced by retinal ischemia/reperfusion through the TLR4/NLRP3 pathway (14). It has been confirmed that puerarin might protect the brain against I/R injury by suppressing autophagy via the AMPK-mTOR-ULK1 signaling pathway (15). However, its effects on renal I/R remain insufficiently explored.

In this study, we investigated whether puerarin could protect the kidney against I/R and determined the potential mechanism and signaling pathway involved in its protective effects.

## Materials and Methods

***Experimental animals and renal I/R model***

This research project received approval from the Research Ethics Committee of Renmin Hospital of Wuhan University, and all experimental procedures strictly adhered to the guidelines for the Care and Use of Laboratory Animals. Ten-week-old adult male Sprague Dawley rats (250-270 g) were sourced from the Experimental Animal Center of the Medical College of Wuhan University (Wuhan, China). Firstly, the rats were completely anesthetized with pentobarbital sodium (50 mg/kg) by intraperitoneal injection and placed on a thermostatic blanket. All rats were randomly allocated to different groups. Subsequently, all rats were exposed to longitudinal abdominal incisions and had their right kidney removed. Then, in the I/R group, the left renal artery was clamped with a non-invasive vascular clamp, and the arterial clamp was removed 45 min later to restore blood supply. At the same time, the incision was sutured without follow-up treatment in the sham group. Following previous protocols, puerarin was dissolved in physiological saline^15^ and administered intraperitoneally once daily for seven days, with 50 and 100 mg/kg16 doses. The sham group, receiving 0 mg/kg of puerarin, was given an equivalent amount of physiological saline. On the 7th day, one hour after puerarin administration, the rats underwent the aforementioned surgical procedure based on their respective groups.

***Cell culture and hypoxia/reoxygenation (H/R) model***

Human renal proximal tubular epithelial cells (HK-2) were obtained from the China Center for Type Culture Collection (CCTCC, Wuhan, China) and cultured in Dulbecco’s modified Eagle’s medium (DMEM) from Invitrogen, United States, supplemented with 10% fetal bovine serum. The incubation environment consisted of 5% carbon dioxide and 95% air, maintaining a temperature of 37 ^°^C. Three days before establishing the hypoxia/reoxygenation (H/R) model, cells were treated with puerarin at concentrations of 1 uM and 10 uM once a day, respectively. In the control group, an equivalent volume of physiological saline was administered to the cells. The H/R model *in vitro* was created following established protocols. HK-2 cells were subjected to a nutrient-free medium for 12 hr under hypoxic conditions (1% O_2_, 94% N_2_, and 5% CO_2_). Next, the medium was replaced with a normal medium, and then the cells were cultured under a normoxic cell incubator (5% CO_2_ and 95% air) for 24 hr. The control group was incubated in a complete culture medium under normoxic conditions.

***Hematoxylin and eosin (H*** ***&*** ***E) staining***

Kidney tissues were fixed in 4% paraformaldehyde and subsequently embedded in paraffin. Sections of 4 μm thickness were prepared for hematoxylin and eosin (H&E) staining. The ischemia/reperfusion (I/R) injury was evaluated using a well-established grading scale, as proposed previously (17).

***Immunohistochemistry and immunocytochemistry***

A Polink-1 one-step polymer detection system (ZSGB-BIO, Beijing, China) was used to perform immunohistochemistry and immunocytochemistry. The kidney section and cell sample were stained with anti-caspase-3 (1:200, Abcam, Ab13847), followed by incubation with secondary antibodies, and then detected with the EnVision/HRP Kit (Dako, Denmark). Each group’s relative mean integrated optical density (IOD) was divided by the average IOD of the control. All sections were photographed at a magnification of 400×.

***Western blot analysis***

Kidney tissue and HK-2 cells were lysed with RIPA buffer (Beyotime, Jiangsu, China) containing protease inhibitors to collect total proteins. The BCA kit (Abcam, Shanghai, China) was used to quantify the total proteins. The protein samples were separated in sodium dodecyl sulfate (SDS) polyacrylamide gel and transferred to polyvinylidene difluoride (PVDF) membranes, which were blocked with 5% skimmed milk. Next, the membranes were immunoblotted with primary antibodies as follows: GRP78 (Abcam, dilution 1:1000); p-elF2α (Abcam, dilution 1:1000); elF2α (Abcam, dilution 1:1000); CHOP (Cell Signaling, dilution 1:1000); Nrf2 (Abcam, dilution 1:1000); HO-1 (Abcam, dilution 1:10000); β-actin (Boster Biological Technology, dilution 1:5000). Subsequently, the membranes were incubated with an appropriate secondary antibody for 2 hr, followed by western blot using the Chemiluminescent HRP. Image Lab Software (NIH, USA) was applied to quantify protein levels.

***Assessment of renal function***

Assessments were carried out using commercial kits, and 2 ml of blood was collected immediately after 24 hr reperfusion from the experimental rats. The kits were employed in accordance with the product instructions (Nanjing Jiancheng Co., China). Serum levels of blood urea nitrogen (BUN) and creatinine (Cr) were calculated by spectrophotometric measurements.

***TUNNEL staining***

An *In Situ* Cell Death Detection Kit, POD (Roche, Germany), was used to perform the TUNNEL staining according to the manufacturer’s protocols. Kidney sections were stained with TUNEL kits to label the nuclei of apoptotic cells. The apoptosis index was calculated as the percentage of TUNEL-positive nuclei relative to the total number of nuclei, as determined by I Image-Pro Plus 6.0 software.

***Hoechst staining***

Hoechst 33342 staining (ThermoFisher, USA) was used to measure cell apoptosis following previously reported methods (18). In brief, cells were treated with a vehicle for 48 hr, then centrifuged and collected in Eppendorf tubes. Then, the cells were fixed with 4% paraformaldehyde and stained with Hoechst 33342 reagent for 10 min at room temperature. A fluorescence microscope was used to observe the frequency of apoptotic cells.

***Statistical analysis***

All data were expressed as mean+standard error of the mean (SEM). Group means were compared using one-way analysis of variance (ANOVA) followed by Tukey’s test for *post hoc* analysis. SPSS version 18.0 (IBM, Armonk, NY, USA) was used for statistical analysis, and differences were considered statistically significant when *P*<0.05.

**Figure 1:**
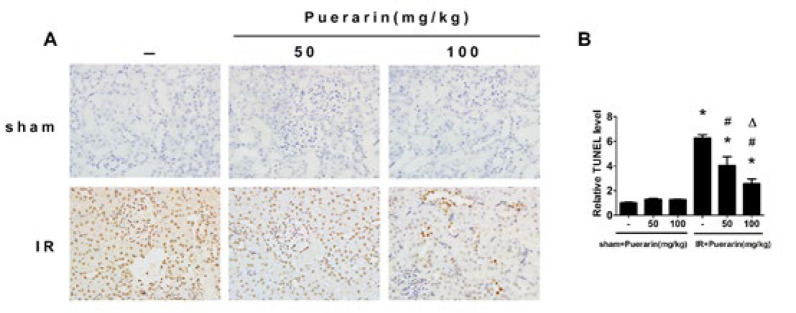
Puerarin alleviated renal ischemia/reperfusion injury in rats

(A) Effect of puerarin at 50 mg/kg and 100 mg/kg on kidney tissue damage detected by H&E (×400). (B-C) Effect of puerarin at doses of 50 mg/kg and 100 mg/kg on serum Cr and BUN levels. (D) Jablonski Grade was used to quantify renal tubular injury (n=8). Values were carried out as the mean±SEM. *P<0.05, relative to the sham group; ^#^*P<*0.05, relative to the IR+puerarin (0 mg/kg) group; ^△^*P<*0.05, relative to IR+puerarin (50 mg/kg) group

**Figure 2:**
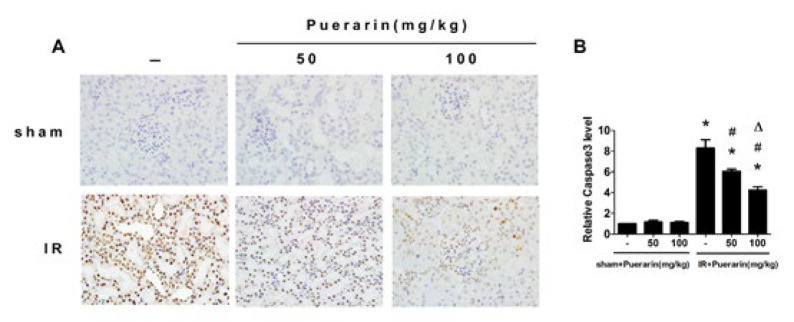
Puerarin reduced apoptosis in rats

(A) Effect of puerarin at doses of 50 mg/kg and 100 mg/kg on numbers of TUNEL-positive cells. (B) Relative Tunnel level was calculated as the percent of TUNEL-positive nuclei relative to total number of nuclei (n=8). Values were carried out as the mean±SEM. *P<0.05, relative to sham group; ^#^*P<*0.05, relative to the IR+sterile saline group; ^△^*P<*0.05, relative to IR +puerarin (50 mg/kg) group

**Figure 3:**
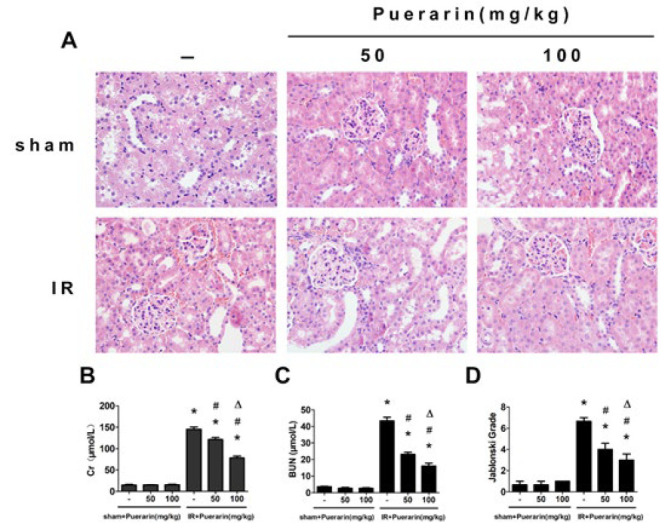
Puerarin inhibit the expression of Caspse-3 in rats

(A-B) Effect of puerarin at 50 mg/kg and 100 mg/kg on Caspse-3 level in rat kidney tissues(n=8). Values were carried out as the mean ± SEM. *P<0.05, relative to sham group; # *P<*0.05, relative to the IR+ puerarin (0 mg/kg) group; ^△^*P<*0.05, relative to IR +puerarin (50 mg/kg) group

**Figure 4:**
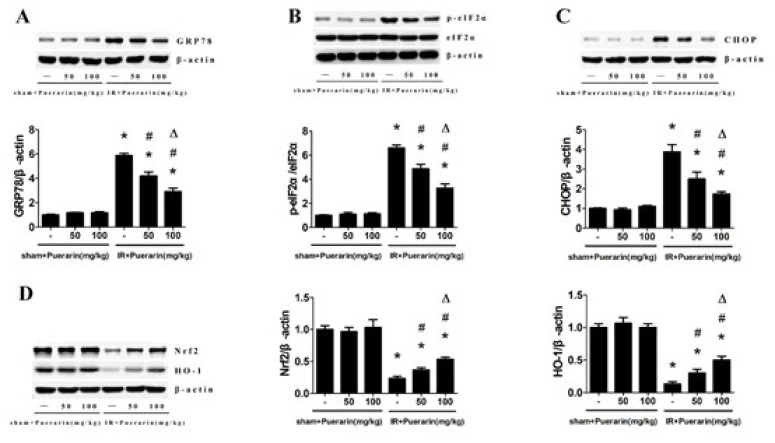
Puerarin inhibited apoptosis and endoplasmic reticulum stress induced by renal I/R in rats

(A-C) Endoplasmic reticulum stress-associated proteins, including GRP78, p-elF2α, and CHOP during renal I/R injury, were down-regulated by puerarin treatment. (D) Western blot analysis of Nrf2 and HO-1 in rats after treatment with puerarin at doses of 50 mg/kg and 100 mg/kg (n=8). Values were carried out as the mean±SEM. *P<0.05, relative to sham group; ^#^*P<*0.05, relative to the IR+ puerarin (0 mg/kg) group; ^△^*P<*0.05, relative to IR+puerarin (50 mg/kg) group

**Figure 5:**
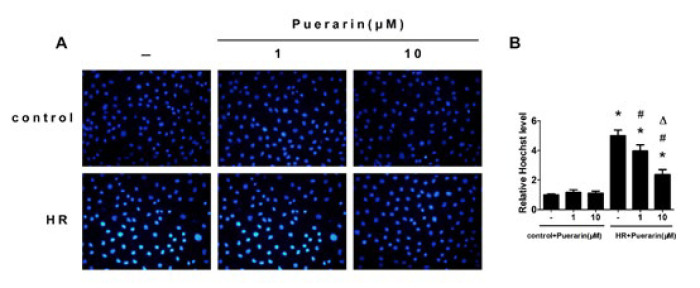
Puerarin reduced apoptosis in HK-2 cells

(A) Effects of puerarin at doses of 50 mg/kg and 100 mg/kg on the frequency of apoptotic cells. (B) Relative Hoechst level was reduced by puerarin treatment in a dose-dependent manner. Values were carried out as the mean ± SEM. *P<0.05, relative to control group; ^#^*P<*0.05, relative to HR +puerarin (0 uM) group; ^△^*P<*0.05, relative to HR+puerarin (1uM) group

**Figure 6:**
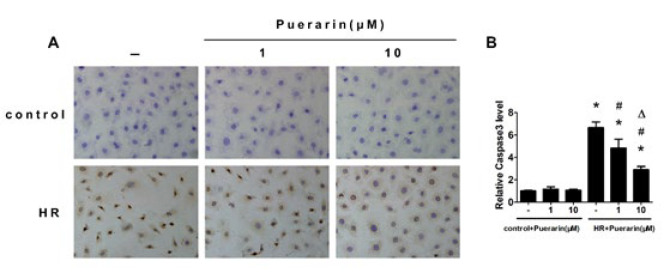
Immunocytochemistry staining of Caspse-3 in HK-2 cells

(A-B) Effect of puerarin at 50 mg/kg and 100 mg/kg on Caspse-3 level in HK-2 cells. Values were carried out as the mean±SEM. *P<0.05, relative to control group; ^#^*P<*0.05, relative to HR+puerarin (0uM) group; ^△^*P<*0.05, relative to HR+puerarin (1uM) group

**Figure 7:**
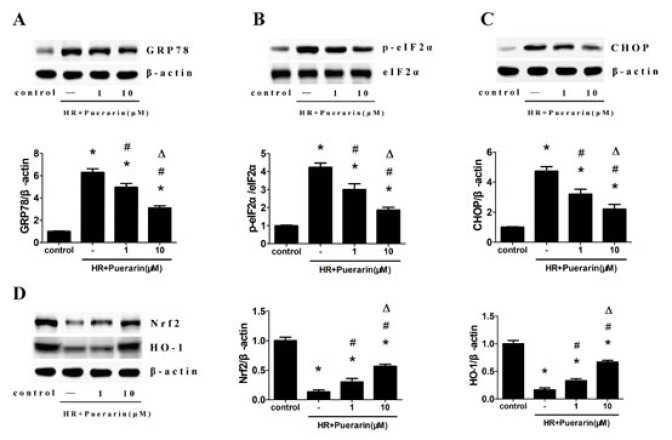
Puerarin inhibited apoptosis and endoplasmic reticulum stress via the Nrf2/HO-1 pathway in HK-2 cells

(A-C) Expression of endoplasmic reticulum stress-associated proteins, including GRP78, p-elF2α, and CHOP after H/R, was down-regulated by puerarin treatment. (D) Western blot analysis of Nrf2 and HO-1 in HK-2 cells at 50 and 100 mg/kg after puerarin treatment. Values are presented as mean±SEM. *P<0.05, relative to the control group; ^#^*P<*0.05, relative to HR+puerarin (0uM) group; ^△^*P<*0.05, relative to HR +puerarin (1 uM) group

## Results

***Puerarin alleviated renal ischemia/reperfusion in vivo***

We established the ischemia/reperfusion (I/R) model and administered the rats with varying concentrations of puerarin. As depicted in Figure 1A, H&E staining verified that puerarin mitigated tubular epithelium dilatation and loss of the brush border. Moreover, compared to the Sham group, rats subjected to I/R exhibited evident renal function impairment, whereas those treated with puerarin demonstrated marked improvement in renal function (Figure 1B-D).

***Puerarin alleviated apoptosis and endoplasmic reticulum stress via the Nrf2/HO-1 pathway in vivo***

To assess whether puerarin could inhibit apoptosis in kidney tissues, TUNEL staining was performed. The results revealed that renal I/R led to a higher number of TUNEL-positive cells compared to the Sham group, which could be markedly eliminated by puerarin treatment (Figure 2). Moreover, immunohistochemistry results for Caspase-3 indicated that I/R enhanced Caspase-3 expression compared to the Sham group, while rats treated with puerarin exhibited a significant reduction in Caspase-3 expression (Figure 3). Next, we performed a Western blot analysis to investigate the potential regulatory effect of puerarin on endoplasmic reticulum stress. Obviously, the expression of endoplasmic reticulum stress-associated proteins, including GRP78, p-elF2α, and CHOP, were noticeably increased after renal I/R. Meanwhile, rats treated with puerarin resulted in a dose-dependent reduction in the expression of these proteins than the I/R group, indicating that puerarin could alleviate I/R-induced endoplasmic reticulum stress (Figure 4A-C). Furthermore, to explore the underlying mechanism, we examined the Nrf2/HO-1 pathway in each group, known to be closely related to endoplasmic reticulum stress and apoptosis. Western blot analysis showed that Nrf2/HO-1 expression was elevated in the I/R group, and this elevation was further enhanced by puerarin treatment (Figure 4D). These findings collectively indicate that puerarin alleviated I/R-induced apoptosis and endoplasmic reticulum stress via the Nrf2/HO-1 pathway *in vivo*.

***Puerarin effectively alleviated apoptosis in H/R exposed HK-2 cells***

The *in vitro* H/R model was constructed further to verify puerarin’s protective effects on renal I/R injury. Hoechst staining demonstrated that H/R exposure would promote HK-2 cells’ apoptosis, and puerarin treatment could relieve this effect (Figure 5). Similarly, as shown in Figure 6, the expression of Caspase-3 was significantly enhanced in HK-2 cells compared with the control group, which was dose-dependently decreased by puerarin treatment.

***Puerarin alleviated endoplasmic reticulum stress via the Nrf2/HO-1 pathway in vitro***

Western blot analysis revealed that the expression of endoplasmic reticulum stress-associated proteins, such as GRP78, p-elF2α, and CHOP, were elevated in the H/R group. Moreover, puerarin could obviously reduce the expression of these proteins in a dose-dependent manner (Figure 7A-C), which was consistent with the conclusions in *in vivo* experiments. Next, western blot analysis indicated that Nrf2/HO-1 expression was elevated in the H/R group compared with the control group, while these changes were reversed by puerarin (Figure 7D). Therefore, our results suggested puerarin could alleviate endoplasmic reticulum stress induced by H/R via the Nrf2/HO-1 pathway *in vitro*. 

## Discussion

According to incomplete statistics, at least 60% of AKI cases are attributed to renal I/R, establishing it as a significant contributor to morbidity and mortality, particularly in intensive care unit (ICU) patients (19, 20). Renal I/R injury is prevalent in many clinical settings, such as major vascular and cardiac surgery, trauma, sepsis, nephrectomy, and kidney transplantation (21). Its pathophysiological pathogenesis is complicated, and available treatment options are often limited and ineffective, underscoring the urgent need to explore and identify new therapeutic targets (22). Our project is designed to investigate whether puerarin has the potential to alleviate renal I/R injury and elucidate the possible underlying mechanisms.

Puerarin, derived from the Chinese medicine Pueraria radix, offers several pharmacological benefits, including reducing oxidative stress, enhancing microcirculation, and improving insulin resistance (23). Many studies have investigated its impact on ischemia-reperfusion injury in various organs (24). For instance, research has shown that puerarin can protect myocardial tissues from ischemia-reperfusion (I/R) injury by up-regulating ANRIL and inhibiting autophagy. Another study suggested that puerarin might inhibit neutrophil-mediated inflammatory response after brain I/R by down-regulating ICAM-1 and suppressing NF-κB activity (25). Our study further confirms puerarin’s protective effect on renal I/R injury. Both in the mouse I/R model and cell H/R model, we demonstrated that puerarin could relieve renal I/R, aligning with the conclusions drawn from I/R injury in other organs. Meanwhile, previous studies have reported that puerarin exhibits protective effects on the kidneys in various diseases such as diabetic nephropathy, obstructive nephropathy, drug-induced kidney injury, and chronic kidney disease. In diabetic nephropathy, puerarin could regulate autophagy to inhibit inflammation and ferroptosis, mitigate podocyte damage caused by oxidative stress, and delay the progression of fibrosis (26-28). In cases of kidney injury induced by multiple drugs, the protective mechanism of puerarin on kidneys includes inhibition of inflammation, oxidative stress, apoptosis, mitochondrial fission, autophagy, and endoplasmic reticulum stress (29-32). Researchers have found that puerarin could increase the expression of renal uptake transporters and promote the excretion of endogenous toxins, indicating that it may be a candidate drug for preventing methotrexate nephrotoxicity (33). Similarly, in obstructive kidney disease and chronic kidney disease, puerarin reverses renal damage by inhibiting inflammation and fibrosis caused by extracellular matrix (ECM) deposition (34, 35). Our study first discovered that puerarin reduces renal damage in ischemia-reperfusion by inhibiting cell apoptosis and endoplasmic reticulum stress.

Apoptosis, the programmed cell death, plays an important role in I/R injury (36). In the context of renal tissue ischemia, endoplasmic reticulum dysfunction ensues, leading to the accumulation of unfolded proteins. This triggers endoplasmic reticulum stress and the subsequent induction of the unfolded protein response (UPR). While UPR serves as a normal adaptive response, it can also foster apoptosis (37). Glucose-regulated protein 78 (GRP78) is pivotal in facilitating protein folding through ATP hydrolysis and is recognized as a negative regulator of UPR (38). Moreover, CHOP and p-eIF2α are also regulation proteins closely associated with endoplasmic reticulum dysfunction during renal I/R injury (39, 40). Our results showed that the expression of these proteins was up-regulated after I/R, and puerarin could significantly prevent this process. Therefore, we conclude that puerarin might protect renal I/R injury by suppressing apoptosis and endoplasmic reticulum stress. According to previous experimental studies, the Nrf2/HO-1 signaling pathway participated in endoplasmic reticulum stress during renal I/R injury (41, 42). Thus, we detected the expression of Nrf2 and HO-1. As expected, the results showed that puerarin’s protective mechanism was possibly related to the activation of the Nrf2/HO-1 signaling pathway.

## Conclusion

In conclusion, we identified puerarin’s protective effect during renal I/R injury. Further, we found that puerarin inhibited apoptosis and endoplasmic reticulum stress induced by renal I/R injury through the Nrf2/HO-1 signaling pathway, which suggests that puerarin might be a potential therapeutic target in the treatment of renal I/R injury.

## Data Availability

The data will be available upon request.

## Declaration

We did not use the AI Tools to write in our manuscript.

## References

[1] Chen J, Xu C, Yang K, Gao R, Cao Y, Liang L (2023). Inhibition of ALKBH5 attenuates I/R-induced renal injury in male mice by promoting Ccl28 m6A modification and increasing Treg recruitment. Nat Commun.

[2] Shao G, He J, Meng J, Ma A, Geng X, Zhang S (2021). Ganoderic acids prevent renal ischemia reperfusion injury by inhibiting inflammation and apoptosis. Int J Mol Sci.

[3] Tang C, Han H, Liu Z, Liu Y, Yin L, Cai J (2019). Activation of BNIP3-mediated mitophagy protects against renal ischemia-reperfusion injury. Cell Death Dis.

[4] Tajima T, Yoshifuji A, Matsui A, Itoh T, Uchiyama K, Kanda T (2019). Beta-hydroxybutyrate attenuates renal ischemia-reperfusion injury through its anti-pyroptotic effects. Kidney Int.

[5] Pan B, Sun J, Liu Z, Wang L, Huo H, Zhao Y (2021). Longxuetongluo capsule protects against cerebral ischemia/reperfusion injury through endoplasmic reticulum stress and MAPK-mediated mechanisms. J Adv Res.

[6] Zhu YL, Huang J, Chen XY, Xie J, Yang Q, Wang JF (2022). Senkyunolide I alleviates renal Ischemia-Reperfusion injury by inhibiting oxidative stress, endoplasmic reticulum stress and apoptosis. Int Immunopharmacol.

[7] Tang C, Hu Y, Gao J, Jiang J, Shi S, Wang J (2020). Dexmedetomidine pretreatment attenuates myocardial ischemia reperfusion induced acute kidney injury and endoplasmic reticulum stress in human and rat. Life Sci.

[8] Zhang Y, Yang X, Ge X, Zhang F (2019). Puerarin attenuates neurological deficits via Bcl-2/Bax/cleaved caspase-3 and Sirt3/SOD2 apoptotic pathways in subarachnoid hemorrhage mice. Biomed Pharmacother.

[9] Gao M, Zhang Z, Lai K, Deng Y, Zhao C, Lu Z (2022). Puerarin: A protective drug against ischemia-reperfusion injury. Front Pharmacol.

[10] Wang ZK, Chen RR, Li JH, Chen JY, Li W, Niu XL (2020). Puerarin protects against myocardial ischemia/reperfusion injury by inhibiting inflammation and the NLRP3 inflammasome: The role of the SIRT1/NF-kappaB pathway. Int Immunopharmacol.

[11] Zheng M, Song D, Luo Z, Lu Z, Wu Y, Wang W (2015). Effect of puerarin on expression of Fas/FasL mRNA in pulmonary injury induced by ischemia-reperfusion in rabbits. Nat Prod Commun.

[12] Zhang Q, Yao M, Qi J, Song R, Wang L, Li J (2023). Puerarin inhibited oxidative stress and alleviated cerebral ischemia-reperfusion injury through PI3K/Akt/Nrf2 signaling pathway. Front Pharmacol.

[13] Ma Y, Gai Y, Yan J, Li J, Zhang Y (2016). Puerarin attenuates anoxia/reoxygenation injury through enhancing Bcl-2 associated athanogene 3 expression, a modulator of apoptosis and autophagy. Med Sci Monit.

[14] Guan L, Li C, Zhang Y, Gong J, Wang G, Tian P (2020). Puerarin ameliorates retinal ganglion cell damage induced by retinal ischemia/reperfusion through inhibiting the activation of TLR4/NLRP3 inflammasome. Life Sci.

[15] Wang JF, Mei ZG, Fu Y, Yang SB, Zhang SZ, Huang WF (2018). Puerarin protects rat brain against ischemia/reperfusion injury by suppressing autophagy via the AMPK-mTOR-ULK1 signaling pathway. Neural Regen Res.

[16] Wu Z, Li C, Li Q, Li J, Lu X (2020). Puerarin alleviates cisplatin-induced acute renal damage and upregulates microRNA-31-related signaling. Exp Ther Med.

[17] Fang X, Lan X, Zhu M, He M, Sun M, Cao Y (2024). Puerarin induces macrophage M2 polarization to exert antinonalcoholic steatohepatitis pharmacological activity via the activation of autophagy. J Agric Food Chem.

[18] Huang Q, Wang L, Ran Q, Wang J, Wang C, He H (2019). Notopterol-induced apoptosis and differentiation in human acute myeloid leukemia HL-60 cells. Drug Des Devel Ther.

[19] Zhang J, Bi J, Ren Y, Du Z, Li T, Wang T (2021). Involvement of GPX4 in irisin’s protection against ischemia reperfusion-induced acute kidney injury. J Cell Physiol.

[20] Stockwell BR, Friedmann Angeli JP, Bayir H, Bush AI, Conrad M, Dixon SJ (2017). Ferroptosis: A regulated cell death nexus linking metabolism, redox biology, and disease. Cell.

[21] Wu R, Li J, Tu G, Su Y, Zhang X, Luo Z (2021). Comprehensive molecular and cellular characterization of acute kidney injury progression to renal fibrosis. Front Immunol.

[22] Zwaini Z, Dai H, Stover C, Yang B (2017). Role of complement properdin in renal ischemia-reperfusion injury. Curr Gene Ther.

[23] Hou N, Huang Y, Cai SA, Yuan WC, Li LR, Liu XW (2021). Puerarin ameliorated pressure overload-induced cardiac hypertrophy in ovariectomized rats through activation of the PPARalpha/PGC-1 pathway. Acta Pharmacol Sin.

[24] Han Y, Wang H, Wang Y, Dong P, Jia J, Yang S (2021). Puerarin protects cardiomyocytes from ischemia-reperfusion injury by upregulating LncRNA ANRIL and inhibiting autophagy. Cell Tissue Res.

[25] Sang HF, Mei QB, Xu LX, Wang Q, Cheng H, Xiong LZ (2004). Effect of puerarin on neural function and histopathological damages after transient spinal cord ischemia in rabbits. Chin J Traumatol.

[26] Zhu Q, Yang S, Wei C, Lu G, Lee K, He JC (2022). Puerarin attenuates diabetic kidney injury through interaction with Guanidine nucleotide-binding protein Gi subunit alpha-1 (Gnai1) subunit. J Cell Mol Med.

[27] Li X, Cai W, Lee K, Liu B, Deng Y, Chen Y (2017). Puerarin attenuates diabetic kidney injury by suppressing NOX4 expression in podocytes. Sci Rep.

[28] Li X, Zhu Q, Zheng R, Yan J, Wei M, Fan Y (2020). Puerarin attenuates diabetic nephropathy by promoting autophagy in podocytes. Front Physiol.

[29] Ma JQ, Ding J, Xiao ZH, Liu CM (2014). Puerarin ameliorates carbon tetrachloride-induced oxidative DNA damage and inflammation in mouse kidney through ERK/Nrf2/ARE pathway. Food Chem Toxicol.

[30] Ma X, Yan L, Zhu Q, Shao F (2017). Puerarin attenuates cisplatin-induced rat nephrotoxicity: The involvement of TLR4/NF-kappaB signaling pathway. PLoS One.

[31] Liu G, Zhang K, Dong W, Tan Y, Long M, Zou H (2020). Puerarin restores the autophagic flux to alleviate cadmiuminduced endoplasmic reticulum stress in NRK52E cells. Mol Med Rep.

[32] Song XB, Liu G, Wang ZY, Wang L (2016). Puerarin protects against cadmium-induced proximal tubular cell apoptosis by restoring mitochondrial function. Chem Biol Interact.

[33] Liu Q, Liu Z, Huo X, Wang C, Meng Q, Sun H (2018). Puerarin improves methotrexate-induced renal damage by up-regulating renal expression of Oat1 and Oat3 in vivo and in vitro. Biomed Pharmacother.

[34] Wang J, Ge S, Wang Y, Liu Y, Qiu L, Li J (2021). Puerarin alleviates UUO-induced inflammation and fibrosis by regulating the NF-kappaB P65/STAT3 and TGFbeta1/Smads signaling pathways. Drug Des Devel Ther.

[35] Yang J, Li B, Wang J, Fan W (2024). Inhibition of pyroptosis of renal tubular epithelial cells by puerarin via regulation of lncRNA NEAT1 ameliorating chronic renal failure. Iran J Kidney Dis.

[36] Liu K, Lan D, Li C, Liu S, Dai X, Song T (2023). A double-edged sword: Role of apoptosis repressor with caspase recruitment domain (ARC) in tumorigenesis and ischaemia/reperfusion (I/R) injury. Apoptosis.

[37] Xu Y, Guo M, Jiang W, Dong H, Han Y, An XF (2016). Endoplasmic reticulum stress and its effects on renal tubular cells apoptosis in ischemic acute kidney injury. Ren Fail.

[38] Shen X, Zhang K, Kaufman RJ (2004). The unfolded protein response--a stress signaling pathway of the endoplasmic reticulum. J Chem Neuroanat.

[39] Porter AW, Brodsky JL, Buck TM (2022). Emerging links between endoplasmic reticulum stress responses and acute kidney injury. Am J Physiol Cell Physiol.

[40] Marciniak SJ, Yun CY, Oyadomari S, Novoa I, Zhang Y, Jungreis R (2004). CHOP induces death by promoting protein synthesis and oxidation in the stressed endoplasmic reticulum. Genes Dev.

[41] Zhang J, Zhang J, Ni H, Wang Y, Katwal G, Zhao Y (2021). Downregulation of XBP1 protects kidney against ischemia-reperfusion injury via suppressing HRD1-mediated NRF2 ubiquitylation. Cell Death Discov.

[42] Wang J, Lu L, Chen S, Xie J, Lu S, Zhou Y (2020). Up-regulation of PERK/Nrf2/HO-1 axis protects myocardial tissues of mice from damage triggered by ischemia-reperfusion through ameliorating endoplasmic reticulum stress. Cardiovasc Diagn Ther.

